# Using an Integrated Social Cognition Model to Explain Green Purchasing Behavior among Adolescents

**DOI:** 10.3390/ijerph182312663

**Published:** 2021-12-01

**Authors:** Amir H. Pakpour, Cheng-Kuan Lin, Mahdi Safdari, Chung-Ying Lin, Shun-Hua Chen, Kyra Hamilton

**Affiliations:** 1 Social Determinants of Health Research Center, Research Institute for Prevention of Non-Communicable Diseases, Qazvin University of Medical Sciences, Qazvin 3419759811, Iran; amir.pakpour@ju.se; 2 Department of Nursing, School of Health and Welfare, Jönköping University, 55111 Jönköping, Sweden; 3 Department of Environmental Health, Harvard T.H. Chan School of Public Health, Boston, MA 02115, USA; chl309@mail.harvard.edu; 4 Department of Environmental Health Engineering, School of Medical Sciences, Tarbiat Modares University, Tehran 1411713116, Iran; m.safdari62@gmail.com; 5 Institute of Allied Health Sciences, College of Medicine, National Cheng Kung University, Tainan 701401, Taiwan; 6 Biostatistics Consulting Center, National Cheng Kung University Hospital, College of Medicine, National Cheng Kung University, Tainan 701401, Taiwan; 7 Department of Occupational Therapy, College of Medicine, National Cheng Kung University, Tainan 701401, Taiwan; 8 Department of Public Health, College of Medicine, National Cheng Kung University, Tainan 701401, Taiwan; 9 School of Nursing, Fooyin University, Kaohsiung 83102, Taiwan; 10 School of Applied Psychology, Menzies Health Institute Queensland, Griffith University, Brisbane, QLD 4122, Australia; kyra.hamilton@griffith.edu.au; 11 Health Sciences Research Institute, University of California, Merced, CA 95343, USA

**Keywords:** adolescence, green purchase, integrated models, health action process approach, theory of planned behavior, social cognition, habit

## Abstract

Strengthening pro-environmental behaviors such as green purchasing behavior is important for environmental sustainability. An integrated social cognition model which incorporates constructs from habit theory, health action process approach (HAPA), and theory of planned behavior (TPB) is adopted to understand Iranian adolescents’ green purchasing behavior. Using a correlational-prospective design, the study recruited Iranian adolescents aged between 14 and 19 years (N = 2374, n = 1362 (57.4%) females, n = 1012 (42.6%) males; Mean (SD) age = 15.56 (1.22)). At baseline (T1), participants self-reported on the following constructs: past behavior; habit strength (from habit theory); action planning and coping planning (from HAPA); and intention, perceived behavioral control, subjective norm, and attitude (from TPB) with respect to green purchasing behavior. Six months later (T2), participants self-reported on their actions in terms of purchasing green goods. Our findings reported direct effects of perceived behavioral control, subjective norms, attitude, and past behavior on intention; intention and perceived behavioral control on green purchase behavior; intention on two types of planning (i.e., action and coping planning); both types of planning on green purchase behavior; and past green purchase behavior and habits on prospectively measured green purchase behavior. These results indicate that adolescent green purchasing behavior is underpinned by constructs representing motivational, volitional, and automatic processes. This knowledge can help inform the development of theory-based behavior change interventions to improve green purchasing in adolescents, a key developmental period where climate change issues are salient and increased independence and demands in making self-guided decisions are needed.

## 1. Introduction

Current ways of living are having adverse effects on the environment on a global scale [1,2,3]. In 2019, levels of greenhouse gases in the atmosphere such as carbon dioxide (CO2) increased to new records [4]. This has presented national governments and world health organizations with wide-scale and complex logistical challenges on how to manage the effects of climate change and minimize the projected environmental costs. In response, the Paris Agreement was adopted in 2015 [5]. The Agreement aims to mobilize a response globally in terms of fighting the threat of climate change and a goal, which was affirmed at the 2021 Glasgow Climate Change Conference (COP 26) [6], of holding the increase in the global average temperature to below 2 °C above pre-industrial levels and of pursuing efforts to limit the temperature increase to 1.5 °C above pre-industrial levels. Consistent with this, The United Nations proposed 17 sustainable development goals [7], targeting climate action in goal 13 [7].

Pro-environmental behaviors that can be performed by individuals at a population level and which can contribute to environmental sustainability and help reduce climate change effects often include behaviors such as reducing red meat consumption, limiting energy consumption, recycling, avoiding waste, and green purchasing [8]. Despite knowledge of these pro-environmental behaviors in contributing to environmental sustainability, the current literature rarely provides evidence for the mechanisms of action that underpin these pro-environmental behaviors. Previous studies have demonstrated a clear gap between consumers’ attitudes toward green purchase products and their actual purchasing behavior. For example, Hughner et al. [8] found 67% of consumers held positive attitudes toward eco-friendly organic food products, yet only 4% purchased these products. This discrepancy demonstrates a clear attitude–behavior gap that has been commonly observed in the literature [9,10,11], and highlights the importance of other factors such as price, convenience, or availability that may influence green purchase behavior [12].

Therefore, further knowledge to inform behavior change interventions that organizations and governments can use to mobilize individuals into performing pro-environmental behaviors is needed. This can be gleaned from extracting principles from literature describing behavioral science methods and models [13]. One emerging line of investigation is in the use of integrated models of behavior (for an overview see [14]). Studies investigating a range of behaviors have shown support for such models in explaining and predicting individuals’ intentions and behavior [15,16,17,18,19,20,21,22,23,24]. These integrated social cognition models are often coined hybrid models, as they draw constructs and specified relations from more than one existing theory to arrive at a new theory or a more comprehensive model. This is because no one theory can be considered definitive in explaining behavior, and thus, should be open to modification to enable other constructs to be added that may provide more efficacious explanations of outcomes such as behavior and the processes involved [14]. One such application has been the integration of constructs from several different well-used psychological theories, including the theory of planned behavior (TPB) [25], health action process approach (HAPA) [26], reflective impulsive model [27], and habit theory [28,29].

The overarching aim of the current study was to test the effectiveness of an integrated model which incorporates constructs that underpin motivational, volitional, and automatic processes in the context of green purchasing behaviors by adolescents. Using more elaborate, comprehensive models of behavior to understand green purchasing behavior among young generations is important as they represent future consumers and can inspire innovative thinking on ideas to reach environmental sustainability [29]. Young consumers are characterized as being more open to new technologies that readily and conveniently support their lifestyles, seeking out more information before purchasing products, and being more likely to translate their intentions to behaviors [29]. Thus, to better understand green purchase behavior among Iranian adolescents, using an integrated social cognition model may provide more comprehensive insights into the social, psychological and behavioral factors that drive this behavior. 

## 2. Overview of Theories in the Proposed Integrated Model of Behavior

### 2.1. Theory of Planned Behavior: Attitude, Subjective Norms, Perceived Behavioral Control, and Intention

Most of the common models and theories used to explain human behavior propose that the dominant predictor of human behavior is intention, and this construct (i.e., intention) is central to TPB [25]. TPB proposes three central predictors of intention; attitude (an individual’s overall evaluation of the target behavior), subjective norms (an individual’s perceived social pressure to carry out the target behavior), and perceived behavioral control (an individual’s perceived capacity and confidence to perform the target behavior, also hypothesized to predict behavior). Meta-analytical findings in general support the effectiveness of TPB in explaining people’s intentions and behavior [30,31]. In relation to pro-environmental behaviors, TPB has successfully explained a range of behaviors including visiting green restaurants and hotels, buying and consuming green products, and purchasing organic products [32,33,34,35,36,37,38,39,40,41,42]. Moreover, the versatility of TPB across cultures, and in adolescent pro-environmental behaviors has also been supported [35,37,38,39,43]. Therefore, TPB motivational constructs of attitude, subjective norms, perceived behavioral control, and intention were included in the current proposed model.

### 2.2. Health Action Process Approach: Action Planning and Coping Planning

The usefulness of TPB in explaining behavior, however, is argued to be limited in its application in terms of understanding the intention–behavior gap. This claim is supported by meta-analytical findings which have found only modest correlations between intention and behavior [44]. Application of dual-phase theories such as HAPA [26,45] have therefore been used to provide an understanding of this knowledge gap. A key feature of the HAPA that makes it distinct from other social cognition models like TPB is that it proposes two phases for behavior generation. Specifically, it proposes a motivational phase and a volitional phase alongside the decisionmaking process; an intention is formed in the motivational phase, and individuals implement their mindset while acting on their intention during the volitional phase [45]. Action planning (a strategy that facilitates a person to perform behavior by making prospective plans concerning how, where, and when to perform the behavior) and coping planning (a strategy that ensures plans are made to overcome obstacles and barriers that may thwart behavioral action) are key constructs in the volitional phase and are considered important determinants of behavior. Behavioral intention is considered to operate as a bridge, linking beliefs that form individuals’ intentions in the motivational phase to self-regulatory strategies (like action and coping planning) in the volitational phase, and thus helping to translate intentions into behavior. Prior research (e.g., [24,45]) has demonstrated support for HAPA constructs in predicting health preventive behaviors, including pro-environmental behaviors [46,47]. Therefore, HAPA volitional constructs of action planning and coping planning were included in the current proposed model.

### 2.3. Automatic Processes: Habit and Past Green Purchasing Behavior

The integration of constructs in TPB and HAPA have often been used to determine the motivational and volitional factors that guide individuals’ intentions and behavior; however, unexplained variance in predicting intentions and behavior remains. Researchers have therefore sought to include constructs that reflect automatic, impulsive processes from dual-process models of action to improve understanding of complex behaviors. For example, the Reflective–Impulsive Model (RIM) [27] proposes two pathways that guide behavior: a deliberative, conscious pathway (underpinned by social cognition constructs in the TPB and HAPA) and an impulsive, non-conscious pathway. The premise behind the latter pathway is that frequently performed behaviors do not require much cognitive effort. This is because the repeated previous experience with the behavior, along with behavioral evaluations that covary with the experience, build up in the mind and lead to more automatic activation of behavior.

One prominent construct reflecting more non-conscious determinants of behavior is habit, defined as a cue–response association, developed over time through the repetition of an action in a stable context [28]. Once a habit is formed, behavioral performance is thought to be guided by ‘automatic’ processes (i.e., non-conscious processes) rather than by conscious, deliberative processes mediated by intention [28,48,49]. Therefore, consistent with dual-process theories of behavior, habit may have a direct effect on behavior. Meta-analytical findings have demonstrated support for the role of behavioral automaticity, an element of habit, in predicting behavior [50], including in the context of pro-environmental behavior [51]. It should also be noted that tests of models of social cognition and integrated models of behavior have often included past behavior in the model for testing its *sufficiency* (c.f., [25]). Moreover, the past behavior–behavior relationship, when shown to mediate the social cognition constructs, may model previous decision making. Further, past behavior may capture non-conscious determinants (e.g., habit) of the subsequent behavior [52]. Although these effects have seldom been tested, several recent studies have reported results concurring with these effects [16,19,53]. Therefore, measures of past green purchase behavior and habit were included in the current proposed model.

## 3. Methods

### 3.1. Participants, Design and Procedure

Participants were adolescents aged 14 to 19 years (M_age_ = 15.56, SD = 1.22) recruited from co-educational high schools in Qazvin, Iran between September 2018 and June 2019. The study adopted a prospective–correlational design with a six-month follow-up. First, members of the research team obtained a list all high schools in Qazvin from the Organization for Education in Qazvin. Next, 33 high schools from the list were randomly selected and within each school three classes across all grades were randomly selected to participate. Six months later, 1859 of the adolescents (78.3% of the baseline sample; n = 1103 (59.3%) female; M_age_ = 15.20, SD = 1.01) completed the follow-up survey. At baseline (i.e., Time 1; T1), participants (N = 2374, n = 1362 (57.4%) female) completed a paper-based questionnaire in class time which assessed demographic factors and the constructs of our proposed model: green purchasing behavior, intention, attitude, subjective norms, perceived behavior control, action planning, coping planning, habit (see Supplementary Material File S1). At Time 2 (T2), participants (N = 1859; n = 1103 (59.3%) female; attrition rate = 21.7% from Time 1) completed a follow-up paper-based questionnaire asking them to report on their green purchasing behavior over the previous six months. Written informed consent from both the adolescent participants and their parents or legal guardians was obtained.

### 3.2. Measures (Supplementary Material File S1)

Measures were developed according to standardized guidelines. In addition, cultural adaptation was taken into consideration when the measures were developed. Finally, psychometric measures with multiple items were used to measure psychological constructs in the present study. Specifically, recently published studies on intention to buy green products among adolescents and youths [37] and predictors of green purchasing behavior [54] were consulted when developing the psychometric instruments used in the present study. Table 1 provides full item descriptions for all measures.

Green purchasing behavior (hereafter, “behavior” indicates green purchasing behavior in the present study; T1 green purchasing behavior assesses past behavior and T2 green purchasing behavior assesses prospectively measured behavior) (Cronbach’s α = 0.79 and McDonald’s ω = 0.83; Table 1) was measured at T1 and T2 using four items, scored on a 7-point Likert scale with responses *never* (1) to *always* (7). 

**Intention** (Cronbach’s α = 0.90 and McDonald’s ω = 0.93; Table 1) to purchase green products was measured according to TPB guidelines using four items, scored on a 5-point Likert scale with responses (1) *strongly disagree* to (5) *strongly agree*.

**Attitude** (Cronbach’s α = 0.84 and McDonald’s ω = 0.88; Table 1) toward purchasing green products was measured according to TPB guidelines using seven semantically differential items. The responses were scored (1) *extremely bad, extremely undesirable, extremely unenjoyable, extremely foolish, extremely* *unfavorable, extremely unpleasant, or extremely unsatisfying* to (5) *extremely good, extremely desirable, extremely enjoyable, extremely wise, extremely favorable, extremely pleasant, or extremely satisfying*. 

**Subjective norms** (Cronbach’s α = 0.77 and McDonald’s ω = 0.79; Table 1) was measured according to TPB guidelines using two items assessing how likely adolescents were to believe important others in their life would want them to purchase products, scored on a 5-point Likert scale with responses (1) *strongly disagree* to (5) *strongly agree*.

**Perceived behavioral control** (Cronbach’s α = 0.83 and McDonald’s ω = 0.86; Table 1) was measured according to TPB guidelines using three items assessing adolescents’ level of self-efficacy and control over purchasing green products, scored on a 5-point Likert scale with responses (1) *strongly disagree* to (5) *strongly agree*.

**Action planning** (Cronbach’s α = 0.83 and McDonald’s ω = 0.87; Table 1) was measured according to HAPA guidelines using four items assessing the extent to which adolescents had made a plan in relation to purchasing green products. All the items begin with the stem: “I have made a plan with details on…” and scored on a 5-point Likert scale with responses (1) *not at all true* to (5) *exactly true*.

**Coping planning** (Cronbach’s α = 0.83 and McDonald’s ω = 0.86; Table 1) was measured according to HAPA guidelines using three items assessing the extent to which adolescents had made a plan to cope with challenging circumstances that may arise to disrupt plans to purchase green products. All the items begin with the stem: “I have made a plan with details on…” and scored on a 5-point Likert scale with responses (1) *not at all true* to (5) *exactly true*.

**Habit** (Cronbach’s α = 0.88 and McDonald’s ω = 0.91; Table 1) was measured using the 12-item Self-Report Habit Index [28]. The 12-item Self-Report Habit Index measures the strength to which purchasing green products were performed habitually by adolescents. All the items begin with the stem: “Purchasing green products is something…” and scored on a 5-point Likert scale with responses (1) *strongly disagree* to (5) *strongly agree*. 

**Demographic variables.** Sex and age (in years) of the participants were self-reported by the adolescents. The highest education level for participants’ father was collected through student school records.

### 3.3. Statistical Analysis

Structural equation modeling (SEM) was applied to examine the hypothesized paths proposed in the integrated social cognition model on green purchasing behavior. The full information maximum likelihood estimator was applied to handle missing data; the standard error of the path estimation was calculated using a bias-corrected bootstrapping approach with 5000 resamples. The data used for analysis in the present study were completely at random, which was supported by Little’s MCAR test (χ^2^ = 308.776, df = 324, *p* = 0.720). Moreover, missing data were <10% and the data were normally distributed (i.e., a value below 3 for skewness and a value below 10 for kurtosis) [55]. In the tested model, measures assessing psychological and behavioral constructs were included as latent variables, and demographics (including participants’ sex, age, and father’s education) were included as observed control variables.

The hypothesized paths included the following hypotheses: In testing the TPB-based motivational paths, we expected attitudes (H1), subjective norm (H2), and perceived behavioral control (H3) would directly predict intentions, and that intentions (H4) and perceived behavioral control (H5) would directly predict behavior. We also expected that attitudes (H6), subjective norms (H7), and perceived behavioral control (H8) would indirectly predict behavior via intentions. In testing the HAPA-based volitional paths, we expected intentions would directly predict action planning (H9) and coping planning (H10). Subsequently, action planning (H11) and coping planning (H12) would directly predict behavior. Further, we expected that intention would predict behavior indirectly via action planning (H13) and coping planning (H14). We also explored whether indirect effects of attitudes (H15), subjective norms (H16), and perceived behavioral control (H17) on behavior via an intention–action planning–coping planning relationship would emerge. In testing the automatic paths, we expected that habit would directly predict behavior (H18). Finally, we expected that past behavior would directly predict behavior (H19), and that indirect non-zero effects of past behavior on behavior mediated by habit (H20) and the TPB and HAPA constructs (H21) would emerge.

Model fit was examined using the χ^2^ test (a nonsignificant test is expected), the comparative fit index (CFI; >0.9 is expected), the Tucker–Lewis index (TLI; >0.9 is expected), the standardized root mean square residual (SRMR; <0.08 is expected), and the root mean square error of approximation (RMSEA; <0.08 is expected). Cronbach’s α (>0.7 is expected), McDonald’s ω (>0.7 is expected), and composite reliability (CR; >0.6 is expected) coefficients were used together to examine the reliability of the study measures (green purchasing behavior, intentions, attitudes, subjective norm, perceived behavioral control, action planning, coping planning, and habit). In addition, the average variance extracted (AVE; >0.5 is expected) was calculated. Moreover, a confirmatory factor analysis using diagonally weighted least squares (DWLS) estimator was constructed to examine all of the studied constructs. Fit indices for the confirmatory factor analysis included standardized root mean square residual (SRMR), RMSEA, CFI, and TLI. The expected values for the fit indices were SRMR < 0.08, RMSEA < 0.08, CFI > 0.9, and TLI > 0.9.

## 4. Results

### 4.1. Participant Characteristics

Adolescents were aged between 14 and 19 years (Mean ± SD age = 15.56 ± 1.22; 57.4% females). The mean education of their father in years was 7.75 ± 3.87. Attrition analyses on participants who completed T1 and T2 and those who completed T1 only indicated no significant differences in age (*F*(1,2178) = 0.072; *p* = 0.789), sex (χ^2^ (1) = 2.014; *p* = 0.169), father’s education level (*F*(1,2162) = 0.580; *p* = 0.447), or psychological variables (Wilks’ λ = 0.996, *F*(10,2153) = 0.801; *p* = 0.628).

### 4.2. Psychometric Properties of the Study Questionnaire

The item properties, internal consistency, and construct validity of the constructs are demonstrated in Table 1. Specifically, the factor loadings were high for every construct (loadings = 0.589 to 0.846 for attitude; 0.724 and 0.792 for subjective norms; 0.802 to 0.864 for perceived behavioral control; 0.852 to 0.899 for intention; 0.627 to 0.863 for past and prospectively measured behavior; 0.746 to 0.851 for action planning; 0.689 to 0.848 for coping planning; and 0.620 to 0.852 for habit). Cronbach’s α and McDonald’s ω were satisfactory for all the constructs (α = 0.77 to 0.90; ω = 0.79 to 0.93), which indicates good internal consistency of the study constructs. Composite reliability (ranged between 0.730 and 0.929) and AVE (ranged between 0.547 and 0.766) were above the recommended cutoffs, which supported the construct validity for the studied constructs. Moreover, a confirmatory factor analysis on the correlated constructs showed satisfactory fit indices (WRMR = 0.899, RMSEA = 0.077, CFI = 0.909, and TLI = 0.901), which further verify the construct validity of the study constructs. The correlations among the study constructs were all significant (ps < 0.001), which supports the criterion-related validity (Table 2). Further, the discriminant validity of the study constructs was checked using comparisons between composite reliability and square root of AVE. Specifically, when the composite reliability is larger than the square root AVE, the discriminant validity of that construct is supported. The discriminant validity for all constructs except for subjective norms was supported (Table 3). 

### 4.3. Structural Model

**Figure 1:**
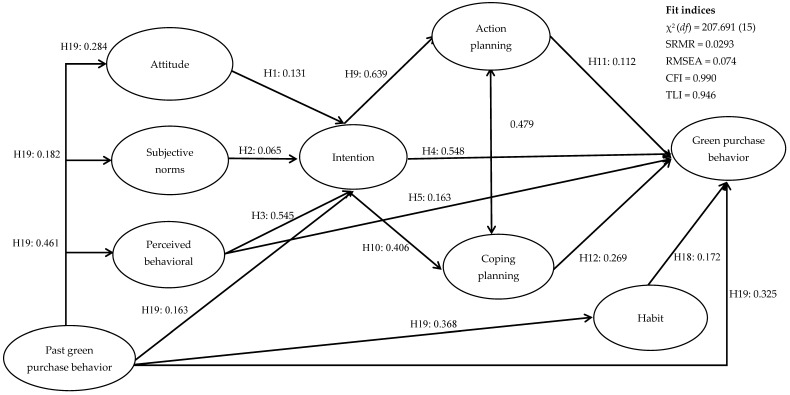
Standardized path coefficients for structural equation model of relations among integrated social cognition model. Note. The following covariates were set to predict all model variables; omitted from the diagram for clarity are age, gender, and father’s education. All hypothesized effects were proposed to be positive in direction.

The structural equation model exhibited adequate model fit with the data (Figure 1) as supported by the fit statistics: SRMR = 0.0293, RMSEA = 0.074, CFI = 0.990, and TLI = 0.946. All path coefficients were significant. Specifically, attitude (H1), subjective norms (H2), and perceived behavioral control (H3) significantly predicted intention to purchase green products. Intention (H4) and perceived behavioral control (H5) significantly predicted prospectively measured behavior. The indirect effects of attitude (H6) and perceived behavioral control (H8) on prospectively measured behavior through intentions were significant, except for subjective norms (H7). Moreover, intention significantly predicted action planning (H9) and coping planning (H10), and action planning (H11) and coping planning (H12) significantly predicted prospectively measured behavior. The indirect effects of intention on behavior through action planning (H13) and coping planning (H14) were significant. Further, the indirect effects of attitude (H15), subjective norm (H6), and perceived behavioral control (H17) on behavior through an intention–action planning–coping planning pathway were significant. Other relevant predictors in the integrated social cognition model also significantly predicted prospectively measured behavior (H18 (habit–behavior) and H19 (past behavior–behavior)). In addition, the indirect effects of past behavior on prospectively measured behavior mediated by habit (H20) and TPB and HAPA constructs (H21) were significant. Inspection of the total effects on prospectively measured green purchasing behavior showed past green purchasing behavior to have the highest association (β = 0.525), followed by perceived behavioral control (β = 0.322), intention (β = 0.313), attitude (β = 0.041), and subjective norms (β = 0.019; Table 4).

## 5. Discussion

We examined the predictions of an integrated social cognition model on adolescents’ green purchasing behavior. The model adopts a priori hypotheses from TPB, HAPA, and dual-process models of action and incorporates constructs that underpin motivational, volitional, and automatic processes. The results indicated that adolescents’ behavior in the purchasing green products was significantly explained by motivational, volitional, and automatic factors. Behavior was significantly explained by intentions and perceived behavioral control, although the direct effects were small, supporting the effects of motivational processes. Behavior was also significantly explained by action planning, coping planning, and habit. Therefore, effects of the constructs underpinning volitional and automatic processes in our proposed model were supported. Moreover, the relative contribution from each process (i.e., motivational, volitional, and automatic) seems to independently affect behavior given that the effects of intentions, perceived behavioral control, planning (including action and coping planning), and habit on behavior were similar. Further, model effects were independent of past green purchasing behavior.

A key contribution of this research was the support found for the multiple pathways by which adolescents’ social cognition affects their green purchasing behaviors. Consistent with motivational components that comprise the deliberative, conscious component of the integrated social cognition model, belief-based factors (e.g., attitude, subjective norms, perceived behavioral control) predicted the green purchase intentions of adolescents. In particular, perceived behavioral control seems to have an especially strong influence over adolescents’ intentions and behavior. This suggests that in addition to using persuasive communication techniques to increase attitudes [11] and promoting the influence of social pressures and norms toward green purchasing [56], techniques to enhance skills and confidence such as modelling, mastery experience, and rewards [57] may be especially important to consider. Adolescents’ green purchasing behavior was explained in this study by the constructs underpinning both volitional and automatic processes, as supported by the significant path coefficients. Moreover, our findings demonstrated that planning (including action and coping planning) and habit had unique contributions in the prediction of green purchasing behavior, and that the effects of intention on behavior were also mediated via planning constructs, as specified in HAPA [26]. This suggests that interventions aiming to improve adolescents’ green purchasing behavior should complement techniques targeting motivation with planning strategies. For example, developing plans that specify the how, where, and when to perform the behavior and developing plans to overcome foreseen barriers and obstacles that may thwart behavioral action [58], as well as environmental restructuring strategies (e.g., developing cues or prompts for individuals to activate implicit beliefs that lead to green purchasing habits or structuring the environment so as to make the wanted behavior easy; [59]).

A further key finding concerned the direct and indirect effects (via social cognition and habit) of habit and past behavior on future behavior. The observed direct effect concurs with previous studies [20,53,60] and is theoretically consistent with the propositions of dual-process theories of behavior [27] that suggest an implicit, automatic pathway to behavior. Thus, past behavior and habit should directly impact on behavior. However, a number of scholars have claimed that the relationship between past and future behaviors effectively represent habits [16,52,61,62,63]. This suggests that an indirect effect should be observed; the effect of past behavior on future behavior should be mediated by habit. Present findings supported this indirect effect, although it should be noted that a direct effect of behavior on future behavior was still noted. 

The present findings add to the extant literature on a key environmental behavior by using an integrated model of social cognition to explain the green purchasing behavior of adolescents. This knowledge can guide policy makers and interventionalists in the design of effective strategies for promoting green purchasing behaviors in adolescent populations, especially at a time where climate change issues are salient. For example, messages may consider providing information about the pros and cons of the behavior to increase attitudes, messages about others’ approval of the behavior to increase subjective norms, and instructions on how to buy green products to increase perceived behavioral control, as well as engaging adolescents in making detailed plans to buy green products and providing cues to action. In this regard, adolescents’ beliefs and habits about purchasing green products may become stronger, and in turn increase their actual green purchasing behavior. 

The present findings, however, should be considered in light of noted limitations. Given the correlational design, inference of causality is not permitted on the basis of the data, only theory. Additionally, the study recruited adolescents who were educated, which may bias the results as young and educated consumers are more likely to be susceptible to socially desirable responses [64]. Furthermore, it may be important to consider other potentially relevant factors identified in the prior literature, such as environmental concern and knowledge [37], and the perceived value and price of green products [65,66]. Moreover, the discriminant validity of the subjective norm construct was somewhat unsatisfactory, although it should be noted that the discriminant validity of all other constructs in the present study was satisfactory. Finally, the present findings were tested among Iranian adolescents; thus, applying the model in other adolescent populations is warranted to ensure replication of findings and generalizability to other cultural groups. Future studies are therefore needed to overcome the aforementioned methodology problems and in so doing add to the current findings to provide strong evidence of the factors that influence green purchasing behavior among adolescents. For example, using objective measures of green purchasing behavior instead of self-reports may overcome issues with social desirability. Assessing other potentially important constructs such as environmental concerns, environmental knowledge, perceived value, and the price of green products may provide further explanation of adolescents’ green purchasing behavior. Further, using a randomized controlled trial design where intervention participants are given a particular strategy vs. a control group could help to outline how behavioral interventions work by demonstrating that specific techniques affect changes in behavior by changing the linked determinants, providing confirmation of the mechanism of action. For example, a poster providing the message that purchasing green products will help the environment (the technique) should affect green purchasing behavior (the behavior) through changing belief about the outcome of performing the behavior (attitude, that is, the mediator).

## 6. Conclusions

We recruited a large sample of Iranian adolescents to examine an integrated social cognition model in the context of green purchasing behavior. Overall, the present findings supported the primary effects of the proposed model (i.e., the motivational, volitional, and automatic factors), suggesting that adolescents’ green purchasing behavior is guided by multiple processes. The findings fill a knowledge gap regarding the utility of using an integrated social cognition model to explain the green purchasing behavior of adolescents. Specifically, the findings suggested that motivational and volitional factors from TPB and HAPA (e.g., perceived behavioral control, planning) as well as automatic factors such as habit have an influence on the green purchasing behavior of adolescents. The present findings can therefore be used to inform the development of future theory-based behavior change interventions to improve green purchasing in adolescents, a key developmental period where climate change issues are salient and where increases in independence and demands to make self-guided decisions are needed.

## Figures and Tables

**Table 1:** Measurement Items and Indicators of Model Fit.

Construct	Measurement Item	λ	α	ω	CR	AVE
Attitude	*When thinking about buying green product, my feeling is…*		0.84	0.88	0.892	0.547
	extremely bad/extremely good	0.811				
	extremely undesirable/extremely desirable	0.667				
	extremely unenjoyable/extremely enjoyable	0.846				
	extremely foolish/extremely wise	0.803				
	extremely unfavorable/extremely favorable	0.589				
	extremely unpleasant/extremely pleasant	0.797				
	extremely undsatisfying/extremely satisfying	0.618				
Subjective Norm	*Most people who are important to me would…*		0.77	0.79	0.730	0.576
	expect me to buy ecofriendly products for personal use.	0.792				
	consider I should buy green products for personal use.	0.724				
Perceived Behavioral Control			0.83	0.86	0.865	0.688
	I can completely make my decision to purchase green product at place of conventional non-green product.	0.864				
	There are resources, time and opportunities for me to purchase green product.	0.802				
	I am confident that I can purchase green product at place of conventional non-green product as long as I want to do so.	0.821				
Intention	*In the near future, I…*		0.90	0.93	0.929	0.766
	am willing to purchase green products.	0.860				
	am willing to buy products with a green mark.	0.852				
	plan to purchase products with a green mark.	0.899				
	will choose products that avoid using corrosive chemical materials.	0.888				
Behavior/ Past Behavior ^a^	*How often do you make a special effort to purchase products that…*		0.79	0.83	0.836	0.565
	are certified as being environmentally safe?	0.842				
	are produced by environmentally-responsible companies?	0.627				
	are packaged in or made out of recycled materials?	0.863				
	come in a refillable container?	0.644				
Action Planning	*I have made a plan with details on…*		0.83	0.87	0.881	0.650
	what to buy (e.g., buying a product that has a certified environmentally-safe or organic stamp).	0.746				
	where to purchase green products.	0.822				
	when to purchase green products.	0.851				
	what preparation I have to do in order to purchase green products.	0.802				
Coping Planning	*I have made a plan with details on…*		0.83	0.86	0.869	0.626
	what to do if something interferes with my plans.	0.774				
	how to cope with possible setbacks.	0.848				
	what to do in difficult situations to act according to my intentions.	0.689				
	how to motivate myself.	0.844				
Habit	*Purchasing green products is something…*		0.88	0.91	0.925	0.508
	I do frequently.	0.715				
	I do automatically.	0.672				
	I do without having to consciously remember.	0.666				
	that makes me feel weird if I do not do it.	0.733				
	I do without thinking.	0.709				
	that would require effort not to do it.	0.677				
	that belongs to my (daily, weekly, monthly) routine.	0.679				
	I start doing before I realize I’m doing it.	0.726				
	I would find hard not to do.	0.852				
	I have no need to think about doing.	0.707				
	that’s typically “me”.	0.768				
	I have been doing for a long time.	0.620				

λ = factor loading; α = Cronbach’s α; ω = McDonald’s ω; CR = composite reliability; AVE = average variance extracted. ^a^ This indicates green purchase behavior. The italics are the item stems for the construct items.

**Table 2:** Pearson Product Correlation Matrix among Study Variables (N = 1822).

	Mean	SD	Attitude	Subjective Norm	Perceived Behavioral Control	Intention	Action Planning	Coping Planning	Past Behavior	Behavior	Habit
Attitude	2.02	0.61	1.00								
Subjective norm	2.29	0.80	0.42 **	1.00							
Perceived behavioral control	2.54	0.98	0.50 **	0.37 **	1.00						
Intention	2.67	1.03	0.43 **	0.34 **	0.61 **	1.00					
Action planning	2.51	0.83	0.34 **	0.31 **	0.54 **	0.55 **	1.00				
Coping planning	2.44	0.88	0.39 **	0.33 **	0.49 **	0.47 **	0.62 **	1.00			
Past behavior ^a^	2.30	1.32	0.28 **	0.18 **	0.46 **	0.46 **	0.49 **	0.58 **	1.00		
Behavior ^b^	2.33	1.02	0.49 **	0.32 **	0.57 **	0.64 **	0.64 **	0.66 **	0.72 **	1.00	
Habit	2.41	1.11	0.25 **	0.05 **	0.37 **	0.43 **	0.33 **	0.35 **	0.37 **	0.51 **	1.00

** *p* < 0.001.^a^ Green purchasing behavior at Time 1.^b^ Green purchasing behavior at Time 2.

**Table 3:** Discriminant Validity of Study Constructs (N = 1822).

	Composite Reliabilty	Square Root of Average Variance Extracted	Dsicriminant Validity
Attitude	0.892	0.740	Supported
Subjective norm	0.730	0.759	Not supported
Perceived behavioral control	0.865	0.829	Supported
Intention	0.929	0.875	Supported
Action planning	0.881	0.806	Supported
Coping planning	0.869	0.791	Supported
Behavior/Past behavior	0.836	0.752	Supported
Habit	0.925	0.713	Supported

**Table 4:** Direct, Indirect, and Total Effects in the Proposed Model Predicting Green Purchase Behavior.

Path	B (SE)	β	LL	UL
Direct effects				
Attitude → intention	0.219 (0.027)	0.131 ***	0.099	0.163
Subjective norm → intention	0.089 (0.02)	0.065 ***	0.043	0.084
Perceived behavioral control → intention	0.564 (0.019)	0.545 ***	0.512	0.577
Perceived behavioral control → behavior	0.131 (0.012)	0.163 ***	0.132	0.190
Intention → action planning	0.567 (0.025)	0.639 ***	0.589	0.670
Intention → coping planning	0.281 (0.021)	0.406 ***	0.361	0.439
Intention → behavior	0.640 (0.173)	0.548 ***	0.159	0.937
Action planning → behavior	1.636 (0.205)	0.112 ***	0.090	0.143
Coping planning → behavior	3.179 (0.241)	0.269 ***	0.216	0.303
Habit → behavior	1.014 (0.067)	0.172 ***	0.158	0.199
Past behavior → behavior	2.666 (0.110)	0.325 ***	0.297	0.356
Indirect effects				
Attitude → intention → action planning	0.124 (0.018)	0.091 *	0.067	0.113
Attitude → intention → coping planning	0.125 (0.018)	0.086 *	0.066	0.108
Attitude → intention → behavior	0.067 (0.028)	0.004 **	0.002	0.008
Attitude → intention → action planning, coping planning → behavior	0.739 (0.117)	0.041 *	0.031	0.052
Subjective norm → intention → action planning	0.050 (0.011)	0.041 *	0.025	0.055
Subjective norm → intention → coping planning	0.051 (0.011)	0.039 *	0.024	0.054
Subjective norm → intention → behavior	0.016 (0.016)	0.001	0.000	0.003
Subjective norm → intention → action planning, coping planning → behavior	0.301 (0.068)	0.019 *	0.011	0.025
Perceived behavioral control→ intention → action planning	0.320 (0.013)	0.376 *	0.349	0.398
Perceived behavioral control → intention → coping planning	0.321 (0.013)	0.357 *	0.324	0.387
Perceived behavioral control → intention → behavior	0.344 (0.091)	0.031 **	0.021	0.046
Perceived behavioral control → intention → action planning, coping planning → behavior	1.906 (0.138)	0.170 **	0.150	0.192
Intention → action planning → behavior	1.243 (0.154)	0.114 **	0.092	0.140
Intention → coping planning → behavior	2.779 (0.317)	0.254 **	0.203	0.298
Intention → action planning, coping planning → behavior	2.739 (0.192)	0.254 *	0.218	0.278
Past behavior → habit → behavior	1.687 (0.113)	0.203 **	0.185	0.226
Past behavior → TPB, HAPA → behavior	3.324 (0.137)	0.434 *	0.407	0.459
Total effects				
Attitude → behavior	0.739 (0.117)	0.041 *	0.031	0.052
Subjective norm → behavior	0.301 (0.068)	0.019*	0.011	0.025
Perceived behavioral control → behavior	3.617 (0.176)	0.322 **	0.295	0.350
Intention → behavior	3.379 (0.229)	0.313 ***	0.278	0.347
Past behavior → behavior	4.353 (0.156)	0.525	0.498	0.552

Note. Age, gender, and father’s education were controlled in the hypothesized model. B = unstandardized path coefficient; SE = standard error; β = standardized path coefficient; LL = lower limit at 95% confidence interval of path coefficient; UL = upper limit at 95% confidence interval of path coefficient; TPB = theory of planned behavior; HAPA = health action process approach. * *p* < 0.05, ** *p* < 0.01, *** *p* < 0.001.

## Data Availability

The data presented in this study are not publicly available due to privacy concerns aligned with our research ethics board. The data will be available upon reasonable request to the corresponding authors.

## Supplementary Materials

The following are available online at https://www.mdpi.com/article/10.3390/ijerph182312663/s1, File S1: Survey questionnaire.
