# Supplementary material for: Using an Integrated Social Cognition Model to Explain Green Purchasing Behavior among Adolescents
Source: Int J Environ Res Public Health. 2021 Dec 1;18(23):12663. doi: 10.3390/ijerph182312663 (PMC8656670; doi:10.3390/ijerph182312663)
Supplement: Supplementary file 1 [file ijerph-18-12663-s001.zip › ijerph-1458942-supplementary.pdf]

Supplementary. Survey questionnaire

1. When thinking about buying green product, my feeling is…
    extremely bad (1)
    bad (2)
    neutral (3)
    good (4)
    extremely good (5)
2. When thinking about buying green product, my feeling is…
    extremely undesirable (1)
    undesirable (2)
    neutral (3)
    desirable (4)
    extremely desirable (5)
3. When thinking about buying green product, my feeling is…
    extremely unenjoyable (1)
    unenjoyable (2)
    neutral (3)
    enjoyable (4)
    extremely enjoyable (5)
4. When thinking about buying green product, my feeling is…
    extremely foolish (1)
    foolish (2)
    neutral (3)
    wise (4)
    extremely wise (5)
5. When thinking about buying green product, my feeling is…
    extremely unfavorable (1)
    unfavorable (2)
    neutral (3)
    favorable (4)
    extremely favorable (5)
6. When thinking about buying green product, my feeling is…
    extremely unpleasant (1)
    unpleasant (2)
    neutral (3)
    pleasant (4)
    extremely pleasant (5)
7. When thinking about buying green product, my feeling is…
    extremely unsatisfying (1)
    unsatisfying (2)
    neutral (3)
    satisfying (4)
    extremely satisfying (5)
8. Most people who are important to me would expect me to buy ecofriendly products for personal use.
    strongly disagree (1)
    disagree (2)
    neutral (3)
    agree (4)
    strongly agree (5)
9. Most people who are important to me would consider I should buy green products for personal use.
    strongly disagree (1)
    disagree (2)
    neutral (3)
    agree (4)
    strongly agree (5)
10. I can completely make my decision to purchase green product at place of conventional non-green product.
    strongly disagree (1)
    disagree (2)
    neutral (3)
    agree (4)

strongly agree (5)

11. There are resources, time and opportunities for me to purchase green product.
    strongly disagree (1)
    disagree (2)
    neutral (3)
    agree (4)
    strongly agree (5)
12. I am confident that I can purchase green product at place of conventional non-green product as long as I want to do so.
    strongly disagree (1)
    disagree (2)
    neutral (3)
    agree (4)
    strongly agree (5)
13. In the near future, I am willing to purchase green products.
    strongly disagree (1)
    disagree (2)
    neutral (3)
    agree (4)
    strongly agree (5)
14. In the near future, I am willing to buy products with a green mark.
    strongly disagree (1)
    disagree (2)
    neutral (3)
    agree (4)
    strongly agree (5)
15. In the near future, I plan to purchase products with a green mark.
    strongly disagree (1)
    disagree (2)
    neutral (3)
    agree (4)
    strongly agree (5)
16. In the near future, I will choose products that avoid using corrosive chemical materials.
    strongly disagree (1)
    disagree (2)
    neutral (3)
    agree (4)
    strongly agree (5)
17. How often do you make a special effort to purchase products that are certified as being environmentally safe?
    never (1)
    seldom (2)
    occasionally (3)
    sometimes (4)
    often (5)
    usually (6)
    always (7)
18. How often do you make a special effort to purchase products that are produced by environmentally-responsible companies?
    never (1)
    seldom (2)
    occasionally (3)
    sometimes (4)
    often (5)
    usually (6)
    always (7)
19. How often do you make a special effort to purchase products that are packaged in or made out of recycled materials?
    never (1)
    seldom (2)
    occasionally (3)
    sometimes (4)

often (5)
usually (6)
always (7)

20. How often do you make a special effort to purchase products that are come in a refillable container
never (1)
seldom (2)
occasionally (3)
sometimes (4)
often (5)
usually (6)
always (7)

21. I have made a plan with details on what to buy (e.g., buying a product that has a certified environmentally-safe or organic stamp).
not at all true (1)
slightly true (2)
somewhat true (3)
true (4)
exactly true (5)

22. I have made a plan with details on where to purchase green products.
not at all true (1)
slightly true (2)
somewhat true (3)
true (4)
exactly true (5)

23. I have made a plan with details on when to purchase green products.
not at all true (1)
slightly true (2)
somewhat true (3)
true (4)
exactly true (5)

24. I have made a plan with details on what preparation I have to do in order to purchase green products.
not at all true (1)
slightly true (2)
somewhat true (3)
true (4)
exactly true (5)

25. I have made a plan with details on what to do if something interferes with my plans.
not at all true (1)
slightly true (2)
somewhat true (3)
true (4)
exactly true (5)

26. I have made a plan with details on how to cope with possible setbacks.
not at all true (1)
slightly true (2)
somewhat true (3)
true (4)
exactly true (5)

27. I have made a plan with details on what to do in difficult situations to act according to my intentions.
not at all true (1)
slightly true (2)
somewhat true (3)
true (4)
exactly true (5)

28. I have made a plan with details on how to motivate myself.
not at all true (1)
slightly true (2)
somewhat true (3)
true (4)
exactly true (5)

29. Purchasing green products is something I do frequently.

strongly disagree (1)
disagree (2)
neutral (3)
agree (4)
strongly agree (5)

30. Purchasing green products is something I do automatically.
    strongly disagree (1)
    disagree (2)
    neutral (3)
    agree (4)
    strongly agree (5)
31. Purchasing green products is something I do without having to consciously remember.
    strongly disagree (1)
    disagree (2)
    neutral (3)
    agree (4)
    strongly agree (5)
32. Purchasing green products is something that makes me feel weird if I do not do it.
    strongly disagree (1)
    disagree (2)
    neutral (3)
    agree (4)
    strongly agree (5)
33. Purchasing green products is something I do without thinking.
    strongly disagree (1)
    disagree (2)
    neutral (3)
    agree (4)
    strongly agree (5)
34. Purchasing green products is something that would require effort not to do it.
    strongly disagree (1)
    disagree (2)
    neutral (3)
    agree (4)
    strongly agree (5)
35. Purchasing green products is something that belongs to my (daily, weekly, monthly) routine.
    strongly disagree (1)
    disagree (2)
    neutral (3)
    agree (4)
    strongly agree (5)
36. Purchasing green products is something I start doing before I realize I'm doing it.
    strongly disagree (1)
    disagree (2)
    neutral (3)
    agree (4)
    strongly agree (5)
37. Purchasing green products is something I would find hard not to do.
    strongly disagree (1)
    disagree (2)
    neutral (3)
    agree (4)
    strongly agree (5)
38. Purchasing green products is something I have no need to think about doing.
    strongly disagree (1)
    disagree (2)
    neutral (3)
    agree (4)
    strongly agree (5)
39. Purchasing green products is something that's typically "me".
    strongly disagree (1)

disagree (2)
neutral (3)
agree (4)
strongly agree (5)

40. Purchasing green products is something I have been doing for a long time.
    strongly disagree (1)
    disagree (2)
    neutral (3)
    agree (4)
    strongly agree (5)
